# Milk Fermented by *Propionibacterium freudenreichii* Induces Apoptosis of HGT-1 Human Gastric Cancer Cells

**DOI:** 10.1371/journal.pone.0031892

**Published:** 2012-03-19

**Authors:** Fabien J. Cousin, Sandrine Jouan-Lanhouet, Marie-Thérèse Dimanche-Boitrel, Laurent Corcos, Gwénaël Jan

**Affiliations:** 1 INRA, Science et Technologie du Lait et de l'Œuf, Rennes, France; 2 AGROCAMPUS OUEST, Science et Technologie du Lait et de l'Œuf, Rennes, France; 3 CNIEL/Syndifrais, Paris, France; 4 Université de Rennes 1, Institut de Recherche Santé Environnement et Travail (IRSET), Rennes, France; 5 Institut National de la Santé et de la Recherche Médicale (INSERM), Team Stress, Membrane and Signaling Rennes, France; 6 Inserm Team, Faculty of Medicine, Brest, France; Vanderbilt University Medical Center, United States of America

## Abstract

**Background:**

Gastric cancer is one of the most common cancers in the world. The “economically developed countries” life style, including diet, constitutes a risk factor favoring this cancer. Diet modulation may lower digestive cancer incidence. Among promising food components, dairy propionibacteria were shown to trigger apoptosis of human colon cancer cells, via the release of short-chain fatty acids acetate and propionate.

**Methodology/Principal Findings:**

A fermented milk, exclusively fermented by *P. freudenreichii*, was recently designed. In this work, the pro-apoptotic potential of this new fermented milk was demonstrated on HGT-1 human gastric cancer cells. Fermented milk supernatant induced typical features of apoptosis including chromatin condensation, formation of apoptotic bodies, DNA laddering, cell cycle arrest and emergence of a subG1 population, phosphatidylserine exposure at the plasma membrane outer leaflet, reactive oxygen species accumulation, mitochondrial transmembrane potential disruption, caspase activation and cytochrome c release. Remarkably, this new fermented milk containing *P. freudenreichii* enhanced the cytotoxicity of camptothecin, a drug used in gastric cancer chemotherapy.

**Conclusions/Significance:**

Such new probiotic fermented milk may thus be useful as part of a preventive diet designed to prevent gastric cancer and/or as a food supplement to potentiate cancer therapeutic treatments.

## Introduction

### Diet modulates the risk of developing gastric cancer

Cancer is the main cause of death in economically developed countries resulting from cancer-associated lifestyle choices including smoking, physical inactivity, and “westernized” diet [1]. Indeed, the role of diet in cancer development is strongly supported by epidemiological studies, in particular regarding cancers of the digestive tract. Gastric cancer (GC) represents the third type of lethal cancers for men in the world. In 2008, 640.600 cases of GC were estimated, causing 464.400 deaths worldwide [1]. This disease is particularly frequent in eastern Asia, and in central and eastern Europe. The most important etiological factors involved in gastric carcinogenesis are diet and *Helicobacter pylori* infection. Substantial evidence, mainly from case-control studies, suggests that the risk of contracting GC is increased by high intake of salted, pickled or smoked traditionally foods, as well as dried fish and meat. On the contrary, fibers, fresh vegetables and fruit, perhaps due to their vitamin C content, were found to be inversely associated with GC risk [2], [3]. The impact of natural/food components on carcinogenesis of the stomach has thus been investigated during the last few decades [4]–[6]. Among these, probiotics and their ability to inhibit the development of cancer attracted scientific interest [7], [8]. A probiotic is generally defined as “a live microorganism which, when administered in adequate amounts, confers a health benefit to the host” [9]. While some microorganisms like *H. pylori* may favor GC, others may have a beneficial effect by preventing digestive cancers. In particular, selected strains of probiotic bacteria can limit cancer development in animal models of carcinogenesis [10]. This was particularly evidenced for colon cancer whereas less is known on the possible effects of probiotics on GC. A possible mechanistic explanation of such a protective effect is the *in situ* release of anti-carcinogenic metabolites such as short chain fatty acids (SCFAs).

### Short chain fatty acids induce apoptosis of cancer cells

SCFA, including acetate, propionate and butyrate, are important anions of the intestinal content and their concentration may be modulated by the diet. They were shown to play a key role in important physiological functions including modulation of the digestive epithelial cells proliferation/apoptosis balance. This involves SCFA-mediated cell-cycle arrest, histone deacetylase inhibition and apoptosis stimulation in transformed cells, but also stimulation of differentiation in healthy cells [11], [12]. It has also been reported that the SCFAs butyrate and propionate both induce apoptosis in human gastric carcinoma cell lines [13]–[15]. Apoptosis is a natural physiologic process allowing cell number regulation and constitutes a target for anti-cancer chemotherapy [16]. Apoptosis allows depletion of cancer cells in a “clean” manner. Cancer cells fragment in a coordinated chain reaction and the residues are absorbed by adjacent tissues and immune system [17], [18]. Two main apoptotic pathways have been described: the extrinsic pathway, which is mediated by activation of death receptors and caspase 8, and the intrinsic pathway, in which mitochondria and caspase 9 are involved [16]. Generally, in cancer cells, the apoptotic function is lacking, which may explain their sustained proliferation and consequently the formation of tumors. The ability to induce apoptosis of cancer cells is thus recognized for its therapeutic potential [16]. Since dairy propionibacteria are able to produce beneficial pro-apoptotic SCFAs, they may prove useful in cancer prevention or treatment.

### *Propionibacterium freudenreichii* can favor apoptotic depletion of digestive cancer cells

This probiotic dairy propionibacterium was shown to induce cell death of different human colon cancer cell lines through apoptosis induction, in co-cultures [19]. The active compounds, secreted into the medium, trigger the intrinsic mitochondrial apoptotic pathway in cultured human colorectal cancer cells. In particular, the SCFAs acetate and propionate released by *P. freudenreichii* induce apoptosis by acting on the mitochondrial adenine nucleotide translocator, causing mitochondria depolarization and permeabilisation, leakage of cytochrome c and caspase activation [19], [20]. However, propionibacterial supernatants are still more pro-apoptotic than the SCFAs acetate and propionate, used at the concentrations measured in supernatants, suggesting that other compounds may be involved in this effect. Selected strains of *P. freudenreichii* remain live and metabolically active within the intestine of humans and of human microbiota-associated rats where they produce SCFAs [21], [22]. Accordingly, they were shown to increase apoptosis in transformed colon epithelial cells of such rats fed with the carcinogen 1,2-dimethylhydrazine (DMH), but not in control healthy ones [23]. Propionibacteria could constitute probiotics favoring colon cancer prophylaxis, provided they reach their target alive and metabolically active. In this context, the vector, or probiotic delivery vehicle, was shown to determine propionibacteria survival and activity [21], [24], [25]. Complex dairy products such as yogurt and cheese, in particular, protect propionibacteria from stress, but contain a complex mixture of bacteria. It is thus not possible to attribute the beneficial effect to a particular strain.

### A fermented milk was evaluated as propionibacteria delivery vehicle in this context

In this study, we used a new food-grade fermented milk containing only *P. freudenreichii* as a sole microflora. As this product contains both live propionibacteria and their active metabolites, it should bring them in contact with the gastric mucosa when ingested. This fermented milk was investigated with respect to HGT-1 human gastric cancer cells apoptosis induction. Indeed, propionibacteria are known to kill human colon cancer cells but their effect on gastric cancer cells is unknown. The fermented milk killed HGT-1 cells in a time- and dose-dependent manner. Typical features of the apoptosis process were observed, including viability drop, chromatin condensation, apoptotic bodies' formation, DNA laddering, ROS production, mitochondrial membrane potential perturbation, caspase activation, cell cycle arrest and emergence of a subG1 population.

## Results

### *P. freudenreichii* fermented milk kills human gastric and colon cancer cells

*P. freudenreichii* ITG P9 grew in 72 h at 30°C in supplemented milk and supplemented milk ultrafiltrate to reach final population of 9,43 log_10_ colony-forming units (cfu)/mL and 9.35 log_10_ cfu/mL, respectively. The SCFA concentrations were 26.21 mM acetate and 54.47 mM propionate (2.08 propionate/acetate ratio) in the milk fermented supernatant whereas they were 24.00 mM and 53.32 mM (2.22 ratio) in the fermented milk ultrafiltrate supernatant. These ratios were expected for propionibacteria, which generally produce twice as much propionate as acetate [26]. Twelve other food-grade dairy propionibacteria strains were also tested (see Table S1 and Figure S1). Using these strains, twelve fermented milks were obtained. Propionate concentrations ranged from 36 to 67 mM and propionibacteria populations from 9.0 to 9.7 log_10_ cfu/mL, for the less and for the most efficient strain, respectively.

**Figure 1:**
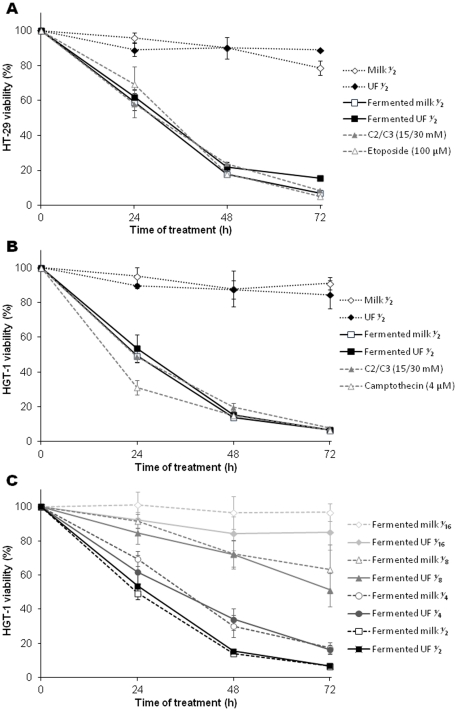
*P. freudenreichii* fermented milk kills human colorectal and gastric cancer cells. (**A**) Effect of *P. freudenreichii* fermented milk on HT-29 human colorectal cancer cells. HT-29 cells were treated with a ½ dilution, in DMEMc, of supernatants obtained from fermented milk or fermented milk ultrafiltrate (squares). As negative controls, cells were treated with ½ dilutions of unfermented milk or milk ultrafiltrate (diamonds). As positive controls (triangles), cells were treated with DMEMc containing a mixture of acetate and propionate (C2/C3, 15/30 mM) or etoposide (100 µM). (**B**) Effect of *P. freudenreichii* fermented milk on HGT-1 human gastric cancer cells. HGT1 cells were treated with a ½ dilution, in DMEMc, of supernatants obtained from fermented milk or fermented milk ultrafiltrate (squares). As negative controls, cells were treated with ½ dilutions of unfermented milk or milk ultrafiltrate (diamonds). As positive controls (triangles), cells were treated with DMEMc containing a mixture of acetate and propionate (C2/C3, 15/30 mM) or camptothecin (4 µM). (**C**) Dose-dependence of fermented milk cytotoxic effect. HGT-1 cells were treated with different dilutions of supernatant obtained from milk (empty symbols) or milk ultrafiltrate (plain symbols) both fermented by *P. freudenreichii*. Cell viability was determined by the methylene blue assay after different periods of treatment. The percentage of viability was calculated using the following formula: 100×(optical density values of treated cells/optical density values of non-treated cells). Results are mean values of three experiments.

The cytotoxic potential of supernatants prepared from milk or milk ultrafiltrate, both fermented by *P. freudenreichii* ITG P9, was investigated. HT-29 human colon cancer cells and HGT-1 human gastric cancer cells were treated with these supernatants. Different dilutions ranging from ½ to 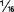 were tested. Moreover, the cytotoxicity of a mixture of 15 mM acetate and 30 mM propionate was tested, at concentrations close to these ½ diluted supernatants. As positive controls of apoptosis induction, the chemotherapy drugs camptothecin (4 µM) and etoposide (100 µM) were used for HGT-1 and HT-29 cells, respectively. Three distinct methods of cell death monitoring were compared. As shown in **Figure S2**, methylene blue, MTT and trypan blue exclusion assays gave similar kinetics of death. Consequently, methylene blue assay was used in this study to further assess cell viability. The non-fermented dairy products (milk or milk ultrafiltrate) had no effect, neither on HT-29, nor on HGT-1 cell viability (**Fig. 1 A,B**). The supernatant of milk or milk ultrafiltrate fermented by *P. freudenreichii* induced a similar cytotoxicity in a time-dependent manner in HT-29 and HGT-1 cells, and close to the cytotoxicity induced by drugs or by the acetate/propionate mixture. Half-maximal killing of HGT-1 cells occurred after 24 h and 26 h of treatment with the fermented milk and fermented milk ultrafiltrate supernatants respectively, after 17 h of treatment with camptothecin and after 24 h of treatment with SCFA mixture (**Fig. S3**). In addition, the cytotoxicity of fermented dairy product supernatants in HGT-1 cells was dose-dependent (**Fig. 1C**). For each dilution tested, the drop in HGT-1 cell viability induced by the fermented milk or the fermented milk ultrafiltrate was very similar (**Fig. 1C** & **Fig. S3**). That's why the next steps of the study we performed only with the fermented milk ultrafiltrate supernatant. Taken together, these data indicate that propionibacteria efficiently kill human colon and gastric cancer cells, at least in part via the production of propionate and acetate (**Fig. 1**). Some other fermented milks, fermented by different dairy propionibacteria strains, showed similar cytotoxic effects on cancer cells (**Table S2**). *P. freudenreichii* ITG P9 being known for its metabolic activity *in vivo* [22], the corresponding fermented milk was further studied.

### *P. freudenreichii* fermented milk induces typical marks of apoptosis in human gastric cancer cells

**Figure 2:**
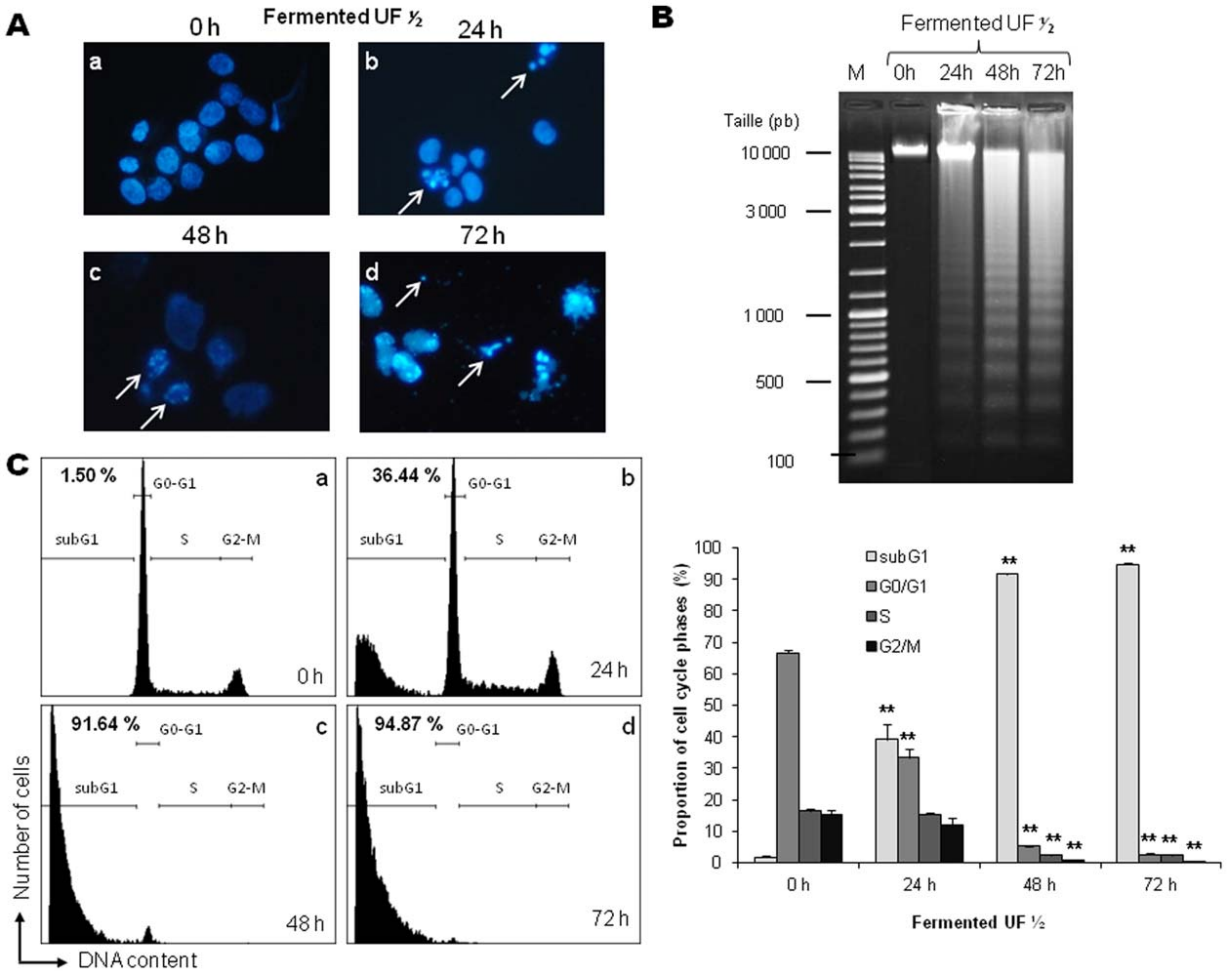
*P. freudenreichii* fermented milk ultrafiltrate induces typical nuclear marks of apoptosis in human gastric cancer cells. (**A**) Fermented milk ultrafiltrate-induced nuclear condensation. Cells were cultured (0 h) or treated for 24, 48 or 72 h with a ½ dilution of *P. freudenreichii* fermented milk ultrafiltrate (UF) supernatant. Cells were then stained with Hoechst 33342 prior to fluorescence microscopy. Arrows indicate chromatin condensation (b), nuclear fragmentation (c) and formation of apoptotic bodies (d). (**B**) Fermented milk ultrafiltrate-induced DNA fragmentation in HGT-1 cells. Genomic DNA was extracted and analyzed in 1% agarose gel. HGT-1 cells were treated as in (A). (**C**) Fermented milk ultrafiltrate-induced changes in cell cycle phase distribution. DNA content of HGT-1 cells was analyzed by flow cytometry after propidium iodide staining. Representative histograms corresponding to DNA content analysis of HGT-1 cells are shown. The percentage of the cell population with sub-G1 DNA content, indicative of apoptosis, is indicated. Proportion of each cell subsets (sub-G1, G0/G1, S, G2/M), within the total cell population, is shown for each time of treatment. Results are mean values of three independent experiments ± sd. *P<0.05, **P<0.01, treated cells versus control (0 h). The distribution of cell cycle phases of untreated cells (control) remained unchanged during the whole experiment.

Nucleus morphology of HGT-1 cells incubated in the presence of *P. freudenreichii* fermented milk ultrafiltrate was analyzed by fluorescence microscopy after Hoechst 33342 staining (**Fig. 2A**). The nuclear fluorescence of untreated cells (control) remained unchanged during the time course of the experiment with blue normal nuclei (time 0 h, **Fig. 2Aa**). During treatment, HGT-1 cells displayed the condensed chromatin and fragmented nuclei characteristics of apoptotic nuclei (**Fig. 2Ab, c**), followed by the appearance of apoptotic bodies at 72 h (**Fig. 2Ad**). Inter-nucleosomal DNA fragmentation resulting in DNA laddering, a typical mark of apoptosis [27], was also monitored during fermented milk ultrafiltrate treatment. DNA fragmentation was observed from 24 h of treatment with fermented milk ultrafiltrate (**Fig. 2B**), and to a lesser extent with camptothecin (**Fig. S3**). DNA content was also quantified by flow cytometry after propidium iodide labeling to study the impact of fermented milk ultrafiltrate on HGT-1 cell cycle distribution (**Fig. 2C**). Untreated HGT-1 cells presented a cell cycle distribution (**Fig. 2Ca**) without subG1 phase, which remained unchanged during the time course of the experiment (data not shown). The percentage of cells in subG1 phase, indicative of an apoptotic process, significantly increased with time during fermented milk ultrafiltrate treatment (**Fig. 2Cb, Cc, Cd**): at 24 h (39.24±4.87%), at 48 h (91.63±0.28%) and at 72 h (94.79±0.45%). At 48 h and 72 h of treatment, all the cells were in the subG1 cell cycle phase, indicating the complete death of HGT-1 cells. As a control, camptothecin induced similar apoptotic hallmarks in HGT-1 cells (**Fig. S4**).

**Figure 3:**
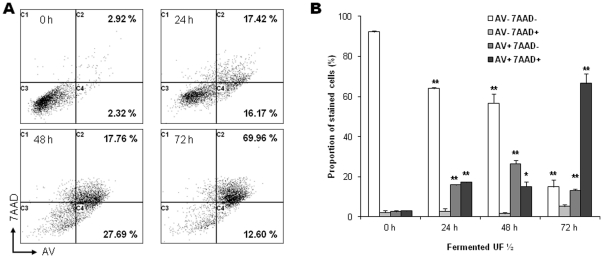
*P. freudenreichii* fermented milk ultrafiltrate induces phosphatidylserine exposure at the plasma membrane outer leaflet in human gastric cancer cells. Flow cytometry kinetic analysis of cell death in HGT-1 cells treated with *P. freudenreichii* fermented milk ultrafiltrate. (**A**) A representative experiment of Annexin V/7-AAD staining of HGT-1 cells at each time of treatment is shown, with proportions of Annexin V positive cells (AV+; apoptotic cells). (**B**) Quantitative FACS analysis of Annexin V-FITC (AV) binding to HGT-1 cells was performed after counterstaining with 7-aminoactinomycin-D (7AAD). Presented values correspond to the proportion of each cell subsets, within the total cell population, for each treatment time. Results are mean values of three independent experiments ± sd. *P<0.05, **P<0.01, treated cells versus control (0 h).

To confirm apoptosis induction, phosphatdylserine translocation from the inner to the outer leaflet of the plasma membrane was assessed by staining HGT-1 cells with a combination of Annexin V-FITC (AV) and 7-AAD, followed by flow cytometry fluorescence analysis (**Fig. 3**). Untreated cells (0 h) presented a high proportion of live cells (AV^−^/7AAD^−^; 92%) and only 5% of cells were stained with Annexin V (**Fig. 3B**). These percentages determined in untreated cells did not change during the time course of the experiment (data not shown). During the treatment of HGT-1 cells with ½ diluted fermented milk ultrafiltrate in DMEMc, the percentage of cells stained only by 7-AAD (indicating necrosis) was very low (**Fig. 3B**). However, the percentages of cells stained with Annexin V increased significantly in a time-dependent manner and reached 80% at 72 h (**Fig. 3B**). Hence, HGT-1 cells underwent apoptosis after treatment with *P. freudenreichii* fermented milk ultrafiltrate, characterized by first AV positive staining, indicating phosphatidylserine exposure, and then both AV and 7-AAD positive staining, indicating a later loss of membrane integrity. As a control, camptothecin induced similar apoptotic hallmarks (**Fig. S5**).

**Figure 4:**
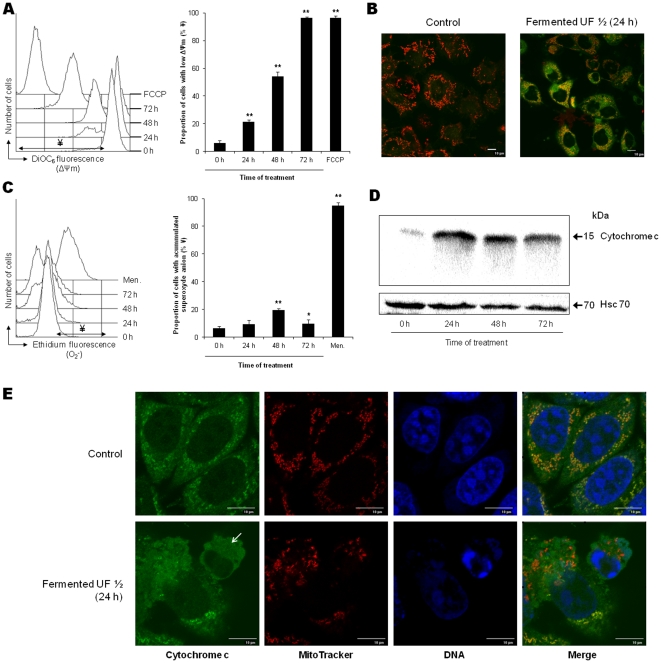
*P. freudenreichii* fermented milk ultrafiltrate induces mitochondrial marks of apoptosis in human gastric cancer cells. Kinetic analysis of mitochondrial events in HGT-1 cells treated with *P. freudenreichii* fermented milk ultrafiltrate (**A**) Flow cytometry kinetic analysis of mitochondrial inner membrane (ΔΨm) dissipation with DiOC(6)3 staining. An overlay view of a representative experiment is shown. The decoupling FCCP (50 µM, 20 min) was used as positive control. Quantitative analyses of ΔΨm loss in 3 independent experiments are shown. Values are represented as a proportion of cells with low ΔΨm, within the total cell population, for each treatment. Results are mean values of three independent experiments ± sd. *P<0.05, **P<0.01, treated versus control (0 h). (**B**) HGT-1 cells were stained with JC-1. The loss of mitochondrial membrane potential is indicated by the progressive loss of red-JC-1-aggregate fluorescence and cytoplasmic diffusion of green monomer fluorescence(**C**) Flow cytometry kinetic analysis of anion superoxide (O_2_^−^) accumulation with DHE staining. An overlay of a representative experiment of ROS detection at each time of treatment is shown. Cells were stained with dihydroethidium and analyzed by flow cytometry. The prooxidant menadione (Men., 100 µM, 15 min) was used as positive control. Values are represented as a proportion of cells with increased ROS (increase of fluorescence intensity), within the total cell population, for each treatment. Results are mean values of three independent experiments ± sd. *P<0.05, **P<0.01, treated versus control (0 h). (**D**) The cytochrome c relocation over the whole treatment period with *P. freudenreichii* fermented milk ultrafiltrate supernatant ½ was assessed by western blot analysis. Following cell treatment, cytoplasm-enrich fractions were analyzed by western blotting and revealed using an anti-cytochrome c antibody. Antibodies directed against Hsc-70 were used as a loading control. (**E**) Subcellular localization of cytochrome c. Cells were immunostained with anti-cytochrome c antibody, following MitoTracker staining and prior to DNA staining using TO-PRO 3. The arrow indicates diffusion of cytochrome c in the cytoplasm.

As SCFA act directly on mitochondria, three important mitochondrial parameters were also determined: the ΔΨm inner membrane potential, and the generation of ROS, using two fluorescent probes, DiOC(6)3 (which is ΔΨm-sensitive) and DHE (which detects O_2_^−^ level), and the localization of cytochrome c. Untreated cells (0 h) exhibited a high fluorescence (high ΔΨm) and a low DHE fluorescence (low production of O_2_^−^) (**Fig. 4A,B**). After treatment with fermented milk ultrafiltrate, a time-dependant decrease in incorporation of DiOC6(3) fluorescent probe was observed, revealing a loss of ΔΨm and hence mitochondrial membrane depolarization (**Fig. 4A**). The percentages of treated HGT-1 cells with decreased inner membrane potential increased in a time-dependent manner to reach 96% of cells at 72 h. FCCP treatment was used as a positive control of mitochondrial membrane depolarization. This loss of ΔΨm was confirmed by the characteristic loss in red fluorescence following JC-1 staining (**Fig. 4B**). Regarding ROS production in HGT-1 cells, fermented milk ultrafiltrate treatment induced accumulation of O_2_^−^-, significantly after 48 h and 72 h of treatment (**Fig. 4C**). This ROS accumulation can be partially prevented if cells are pre-treated by the ROS scavenger TEMPOL (**Fig. S6**). Cytochrome c release from mitochondria to the cytoplasm is a key cellular event of the apoptotic program. Immunoblotting examination of the cytoplasm-enriched fractions for the presence of cytochrome c confirmed a change in its subcellular localization (**Fig. 4D**). Cytochrome c was detected in the cytoplasm-enriched fraction from 24 h of treatment, indicating relocation of this protein (**Fig. 4D**). This relocalization of cytochrome c in the cytoplasm was confirmed by immunostaining, showing diffuse immunostaining throughout the cell after treatment, while punctuated staining before treatment (**Fig. 4E**).

### *P. freudenreichii* fermented milk activates caspases

**Figure 5:**
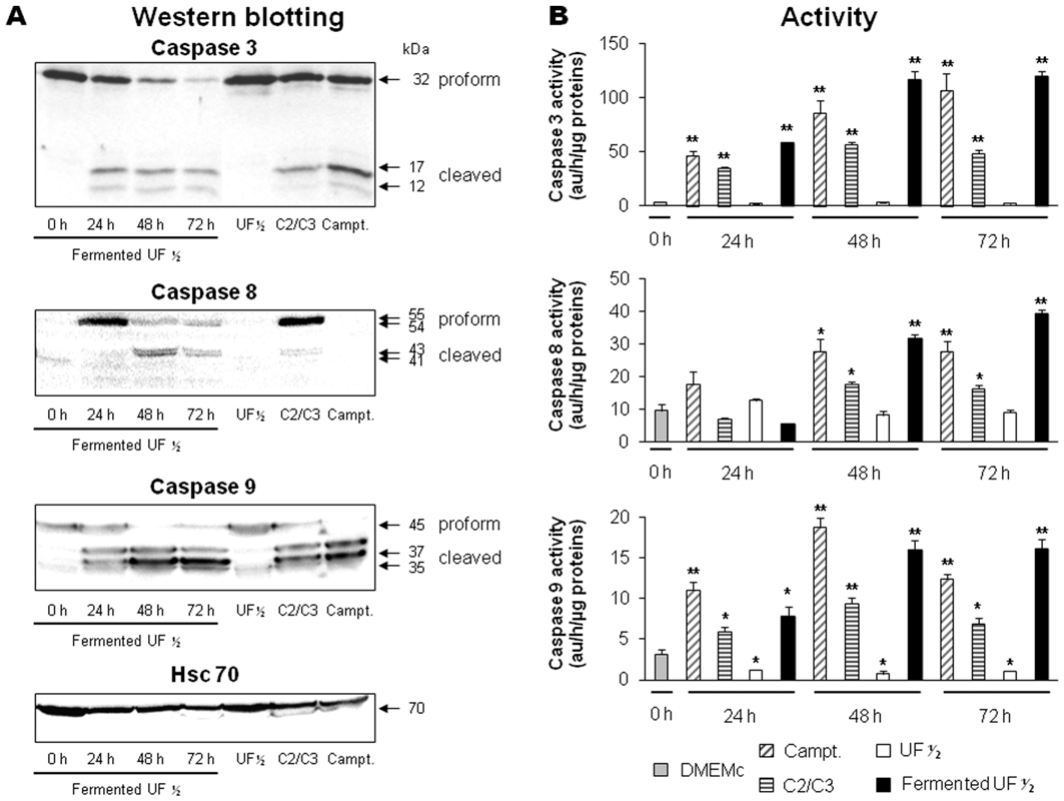
*P. freudenreichii* fermented milk ultrafiltrate activates caspases in human gastric cancer cells. Processing of effector caspase 3 and initiator caspases 8 and 9 was followed by western blotting and specific enzymatic activities monitoring in HGT-1 cells. (**A**) The proforms and cleaved subunits of caspase 3, 8, and 9 were detected by western blotting in lysates of HGT-1 cells treated with *P. freudenreichii* fermented milk ultrafiltrate supernatant ½. Non-fermented milk ultrafiltrate (UF ½, 48 h), a mixture of acetate and propionate (C2/C3, 15/30 mM, 48 h) and camptothecin (4 µM, 48 h) were used as controls. Antibodies directed against Hsc-70 were used as a loading control. (**B**) Caspases specific activities of lysates from cells treated as above were studied using indicated peptides and calculated as described in materials and methods. Results are mean values of three experiments ± sd. *P<0.05, **P<0.01, treated versus control (0 h).

To confirm the apoptotic mechanisms induced by fermented milk ultrafiltrate in HGT-1 cells, processing of caspases 3, 8 and 9 was analyzed by western blotting and corresponding enzymatic activities monitoring in HGT-1 cell extracts (**Fig. 5**). The non-fermented milk ultrafiltrate did not induce caspase activation in HGT-1 cells: only the proforms of caspase-3 and -9 were detected on western blot (**Fig. 5A**) and no caspase activity was measured (**Fig. 5B**). The fermented milk ultrafiltrate supernatant, as well as the camptothecin or the mixture of propionate and acetate in a [2∶ 1] molar ratio, induced the cleavage of pro-caspase 3 and the generation of the active forms of caspase-3, the subunits p17 and p12 (**Fig. 5A**). The activation of caspase-3, which is an effector caspase, confirmed the apoptosis induction. In addition, fermented milk ultrafiltrate induced cleavage of procaspase-9, leading to the 35 and 37 kDa processed forms. Caspase-8 was not detected in control untreated HGT-1 cells. However, treatment with propionibacterial supernatants or C2/C3 mixture led to a clear detection of the 54 kDa proform, followed by its cleavage after 48 h of treatment.

The quantification of the caspase-3, -8 and -9 enzymatic activities confirmed caspase activation by both propionibacterial metabolites and by camptothecin. Indeed, caspases-9 and -3 were activated at the earliest stages of the treatment, while caspase-8 seemed activated later (**Fig. 5B**). Camptothecin and the SCFA mixture activated all the three studied caspases. These data confirmed the activation of the intrinsic pathway of apoptosis by fermented milk ultrafiltrate treatment, with an early activation of caspase-9 and then of caspase-3 and -8.

### *P. freudenreichii* fermented milk increases camptothecin cytotoxicity

**Figure 6:**
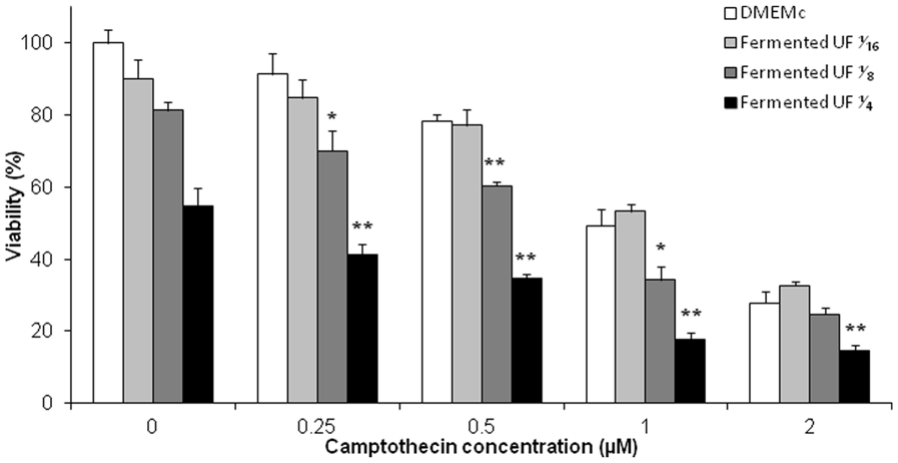
*P. freudenreichii* fermented milk enhances camptothecin cytotoxicity in human gastric cancer cells. Viability of HGT-1 human gastric cancer treated during 24 h was assessed by methylene blue assay. HGT-1 cells were treated with different concentrations of camptothecin (0 to 2 µM) together with different dilutions of *P. freudenreichii* fermented milk ultrafiltrate (UF) supernatant. The percentage of viability was calculated by the following formula: 100×(optical density values of treated cells/optical density values of non-treated cells). Results are mean values of three experiments ± sd. *P<0.05, **P<0.01, treated cells versus controls (same camptothecin concentration without fermented milk ultrafiltrate supernatant and same fermented milk ultrafiltrate supernatant dilution without camptothecin).

Campthotecin is a cytotoxic quinoline alkaloid which inhibits the DNA enzyme topoisomerase I (topo I) and is widely used in the chemotherapeutic treatment of GC. We have shown above that propionibacterial metabolites induce apoptosis in HGT-1 cells via the mitochondrial death pathway. We next studied apoptosis induced by the combination of both camptothecin and fermented milk ultrafiltrate in HGT-1 cells. Increased concentrations of camptothecin (0, 0.25, 0.5, 1, 2 µM) in combination with increased concentrations of fermented milk ultrafiltrate (0, 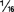, ⅛, ¼) were tested on HGT-1 cell viability and both induced loss of HGT-1 viability, in a dose-dependent manner (**Fig. 6**). Moreover, the addition of fermented milk ultrafiltrate enhanced significantly the cytotoxicity of camptothecin in HGT-1 cells (**Fig. 6**). For example, 0.25 µM camptothecin and fermented milk ultrafiltrate diluted to ⅛ led to a viability loss of 8.6% and 18.6%, respectively. The combination of these two compounds led to a cell viability loss of 29.8% (**Fig. 6**), suggesting an added cytotoxicity effect when camptothecin was combined with fermented milk ultrafiltrate in HGT-1 cells. Accordingly, the combination index (CI), calculated as previously [28], has a value of 1, indicating an additive effect of camptothecin and fermented milk ultrafiltrate treatments.

## Discussion

A new fermented milk, containing *P. freudenreichii* as a sole bacterium, was developed in order to further investigate the probiotic potential of this dairy preparation of propionibacteria (reviewed in [29]). In this study, we report that the aqueous phase of this fermented milk kills human colon and gastric cancer cells via metabolites, including propionate and acetate, released by this bacterium. This notion is based on the observed cytotoxic effects of *P. freudenreichii* fermented milk supernatants on cultured HT-29 and HGT-1 cancer cells. This effect was obtained with ultracentrifugation supernatants, i.e. the fermented milk aqueous phase, devoid of caseins and of bacteria, showing that the active compounds are secreted, as previously described for propionibacteria [19]. For this reason, most of the experiments in this work were performed with fermented milk ultrafiltrate (**Fig. 1**). We demonstrated for the first time that *P. freudenreichii* fermented milk supernatant induced apoptosis of HGT-1 cells in a time- and dose-dependent manner with the appearance of classical morphological and biochemical features of apoptosis (condensed and fragmented chromation, DNA laddering and accumulation of cells in subG1 cell cycle phase).

We looked then if the cellular mechanisms involved in HGT-1 cell death pathway were similar to the mitochondrial death pathway triggered in Caco-2 and HT-29 human colon cancer cells by *P. freudenreichii* DMEMc culture supernatants [19], [20]. The sub-cellular and biochemical events observed in treated HGT-1 cells with fermented milk ultrafiltrate were similar and included, at least, mitochondrial ΔΨm loss, ROS (O_2_^−^) generation, caspases processing and activation, cytochrome c relocation into the cytoplasm (**Fig. 3** **,** **4** **,** **5**). Fermented milk supernatant induced processing and activation of caspase-8, in addition to caspases -3 and -9. Moreover, **Figure 4A** indicates that caspase-8 is not detected in control untreated cells and in cells treated with camptothecin or non-fermented milk ultrafiltrate, in line with the lack of its mRNA (C. Le Jossic-Corcos, Faculty of Medicine, Brest, France, personal communication). It should be noticed that an apparent discrepancy exists between enzymatic results, suggesting caspase-8 activity in camptothecin-treated cells, while no caspase-8 is detected in these cells by western blotting. This can be explained by the lack of specificity of the Ac-IETD-AMC substrate, which can be processed by other enzymes such as caspases -3 and -10 or granzyme B, especially if the concentration of such enzymes is elevated [30], [31]. By contrast, *P. freudenreichii* fermented milk ultrafiltrate and the mixture of SCFA both caused expression and activation of caspase-8. The mechanisms involved here are not elucidated; however, caspase-8 is known to be under-expressed in some cancer cells as a result of DNA methylation [32], [33]. Its expression may be restored at the RNA and protein level by demethylating agents such as decitabine [34] and HDAC inhibitors [35], resulting in demethylation of the regulatory sequence of caspase-8, an increase in caspase-8 promoter activity and in re-expression of caspase-8 [32]. SCFA, such as propionate, are HDAC inhibitors, leading to hyperacetylation of histones [36]. This favors transcription via a change in DNA conformation [37] and could restore the caspase-8 expression. Other food-born compounds were already shown to restore caspase-8 expression. An injectable extract of *Semen coicis* (a relative of maize) extract displaying anti-tumor activity enhances expression of caspase 8 and induces apoptosis in HepG2 cells [38]. Diallyl disulfide, found in garlic, enhances expression of caspase-8, Fas and FasL in leukemia K562 cells. Such a regulation may thus be involved in the pro-apoptotic effects of dairy propionibacteria and of fermented products containing them. Western blotting evidenced restored expression and then late activation of caspase-8 in propionibacterial metabolites-treated HGT-1 cells, which may occur as a result of caspase-8 activation by an another caspase such as caspase-9. Indeed, exit of cytochrome c, part of the mitochondrial pathway, was shown to activate several caspases including 2, 3, 6, 7, 8 and 10 [39]. Furthermore, caspase-9 activation was shown result in down-stream caspase-8 activation [40]. Accordingly, caspase-9 was shown *in vitro* to activate caspase-3 which in turns activates caspase-6, responsible for caspase-8 activation [41]. Altogether, our results confirm that propionibacterial metabolites present in *P. freudenreichii* fermented milk induce several sub-cellular mechanisms favoring apoptosis. Such a reactivation of caspase-8 expression could lead to a major apoptotic response in HGT-1 cells treated with dead domain receptors agonists (e.g. Trail or Fas).

To our knowledge, this is the first work reporting specific induction of gastric cancer cell apoptosis by a fermented dairy product. Fermented milks, including yogurt, were proposed to be useful in the prevention of colon cancer. Epidemiological data on the protective effects of milk and dairy products on colon cancer rate gave conflicting results [42]. Milk and total dairy products consumption is associated with a reduction in colorectal cancer risk. But this is not true for all sorts of dairy products and effects vary greatly. Fermented dairy products may constitute efficient delivery vehicles to target protective molecules or microorganisms to specific sites such as the digestive epithelium [43]. Bovine lactoferrin, a constituent of milk, induces apoptosis of colon [44] and gastric [45] cancer cells. Selected strains of microorganisms present in fermented milks may also counteract colon carcinogenesis. Accordingly, yogurt and lactic acid bacteria exhibited anti-cancer properties in animal models of colon carcinogenesis [46]. However, little work focused on fermented dairy products and gastric cancer. Our work suggests that *P. freudenreichii* could play a role in this context, as it would deliver both live propionibacteria and pro-apoptotic metabolites to the gastric mucosa. In this context, it should be noticed that *P. freudenreichii* adheres to digestive epithelial cells and to mucus [47], [48]. It was also shown to inhibit adhesion of the causative agent of gastric cancer *Helicobacter pylori* to digestive epithelial cells, as well as *H. pylori*-induced damages [49]. These properties suggest a role of *P. freudenreichii* fermented milk in the prophylaxis of gastric cancer.

Finally, our fermented milk potentialized the pro-apoptotic action of the drug camptothecin on gastric cancer cells. Accordingly, live cultures of probiotic lactobacilli were previously shown to sensitize LS513 colorectal cancer cells to 5-fluorouracyl [50]. However, in this case, the probiotics alone had no proapoptotic effect. In this work, low doses of the drug camptothecin used in gastric cancer chemotherapy were inefficient in killing gastric cancer cells. However, in the presence of low doses of *P. freudenreichii* fermented milk, they killed the same cells efficiently (**Fig. 6**). Propionibacterial metabolites induce the intrinsic apoptosis pathway by acting directly on mitochondria, while camptothecin acts at the DNA level. This is consistent with their cumulative potential in apoptosis induction when these pro-apoptotic inducers are combined. Moreover, the fermented milk contains SFCAs, known as HDAC inhibitors able to modulate apoptosis and cell cycle in gastric cancer cells [13], [51] as well as the expression of cell cycle related and apoptotic proteins in other cancer cells [52], [53]. Probiotics were already used in clinical studies to reduce the side effects of cancer chemotherapy [54]. *P. freudenreichii* may, according to our results, be used in such clinical studies. Its synergistic effect may help to lower the drug dose and improve the comfort of patients.

In conclusion, a new milk, exclusively fermented by *P. freudenreichii*, induced apoptosis in HGT-1 human gastric cancer cells. In addition, this fermented milk enhanced the camptothecin cytotoxicity activity, drug used in gastric cancer chemotherapy. Such new probiotic fermented milk might be of interest as a functional food to prevent gastric cancer and/or to potentialize therapeutic treatments.

## Materials and Methods

### Chemicals

Camptothecin, etoposide, TEMPOL, acetate and propionate sodium salts, and caspase substrates Ac-DEVD-AMC (N-Acetyl-Asp-Glu-Val-Asp-7-amido-4-methylcoumarin), Ac-IETD-AMC (N-Acetyl-Ile-Glu-Thr-Asp-7-Amido-4-methylcoumarin) and Ac-LEHD-AFC (N-Acetyl-Leu-Glu-His-Asp-7-amido-4-trifluoromethylcoumarin) were purchased from Sigma-Aldrich (Saint-Quentin Fallavier, France). Camptothecin was dissolved in DMSO. The final concentration of DMSO did not exceed 0.01%, a concentration that did not induce any toxicity. RNAse A, PI (propidium iodide), Hoechst H33342, MitoTracker® Red CMXRos, TO-PRO-3, JC-1, 3,3′-dihexyloxacarbocyanine iodide (DiOC6(3)) and dihydroethidium (DHE) were obtained from Invitrogen (Cergy-Pontoise, France). Annexin V-FITC (AV) kit and 7-aminoactinomycin-D (7-AAD) solution were provided by BD Biosciences (Pont-de-Claix, France). Complete mini protease inhibitor cocktail was obtained from Roche Applied Science (Meylan, France). Mouse antibodies to caspase 8 (clone 12F5, Alexis Biochemicals, Covalab, Villeurbanne, France), cytochrome c (clone 7H8.2C12, BD Biosciences) and Hsc-70 (clone B6, Santa Cruz Biotechnology, Tebu-bio, Le Perray en Yvelines, France) and rabbit antibodies to caspase 3 (clone H-277, Santa Cruz) and caspase 9 (Cell Signalling Technology, Ozyme, Saint-Quentin-en-Yvelines, France) were used for western blots. Polyvinylidene difluoride (PVDF) membranes, horseradish peroxidase (HRP) conjugated secondary antibodies and ECL plus kit were purchased from GE Healthcare (Saclay, France). Sheep antibody to cytochrome c (Sigma-Aldrich) and donkey anti-sheep IgG FITC-linked antibody (Sigma-Aldrich) were used for confocal fluorescence microscopy.

### Bacterial culture and supernatant preparation

The *Propionibacterium freudenreichii* ITG P9 strain (Institut Technique du Gruyère, Actilait, Rennes, France) used in this study was provided by the CIRM-BIA (BIA138, see Table S1 formerly labeled TL133, Centre International de Ressources Microbiennes - Bactéries d'Intérêt Alimentaire, INRA, Rennes, France). It was routinely cultivated at 30°C without shaking in milk ultrafiltrate (UF) supplemented with 100 mM of food-grade sodium lactate (>97% L-lactate, galaflow SL 60, Société Arnaud, Paris, France) and 10 g/L of casein hydrolysate (Organotechnie, La Courneuve, France), sterilized by 0.2 µm filtration (Nalgene, Roskilde, Denmark). To prepare experimental fermented milk, UHT milk (skimmed milk, UHT, Agrilait, Cesson-Sévigné, France) or milk ultrafiltrate (obtained as previously described [55]) was supplemented with 50 mM sodium lactate and 5 g/L casein peptone, inoculated (1%) with *P. freudenreichii* and incubated during 3 days at 30°C. After growth, bacterial supernatants were prepared by centrifugation (12 000× *g*, 4°C, 15 min) for fermented milk ultrafiltrate or by ultra-centrifugation (100 000× *g*, 4°C, 1 h) for fermented milk, neutralized at pH 7 and filter-sterilized as above. The bacterial concentration was determined by CFU counting on YEL agar before centrifugation and production of propionate and acetate was quantified in supernatants by HPLC as previously described [19].

### Cell lines and culture conditions

The human gastric cancer cell line HGT-1 was a kind gift of Dr Laboisse (INSERM, Nantes). The HT-29 human colon adenocarcinoma cell line was obtained from ATCC (American Type Culture Collection, Rockville, MD). Cell lines were was cultured at 37°C under a humidified atmosphere of 5% CO_2_ in DMEM medium (GlutaMAX™, high glucose, Gibco-Invitrogen) supplemented with 10% heat inactivated-fetal calf serum (PAN, Dominique Dutscher, Brumath, France) and antibiotics [56].

### Cell treatments

HGT-1 cells were plated with DMEMc (DMEM with inactivated-fetal calf serum) without antibiotics during at least one night for attachment. When cells reached 75% confluence, the medium was replaced with fresh medium (control) or with treatment media. To treat cells, non-fermented milk ultrafiltrate or milk, and supernatants of milk ultrafiltrate or milk both fermented by *P. freudenreichii* were diluted, from two (½) to sixteen times (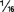), in DMEMc. To study the impact of propionibacterial SCFA, DMEMc supplemented with 15 mM acetate and 30 mM propionate, as previously quantified in the *P. freudenreichii* DMEMc supernatants [19], was used. As a positive control of apoptosis induction, cells were also treated with 4 µM camptothecin (HGT-1) or 100 µM etoposide (HT-29), an anticancer drug known to induce apoptosis [57]. Treatments were performed during 24 h, 48 h and 72 h.

### Cell viability monitoring

Viability of HGT-1 cells was assessed by methylene blue assay as previously described [58]. The percentage of viability was calculated by the following formula: 100×(optical density values of treated cells/optical density values of non-treated cells).

### DNA labeling and fluorescence microscopy

HGT-1 cells were trypsinized, harvested and resuspended in phosphate-buffered saline (PBS) with 1.6 mM of the DNA intercalating Hoechst H33342 compound. After a 30-min incubation at 37°C in the dark, cells were observed under an Optiphot fluorescence microscope (Nikon, Champigny sur Marne, France). Numerical pictures were obtained with a D300 camera (Nikon, Champigny sur Marne, France).

### Confocal microscopy

For potential membrane mitochondria observation, cells were cultured on compartmented glass coverslips before treatment. Following treatment, cells were stained with the JC-1 probe (10 µg/mL, 30 min, 37°C) as described previously [59].

For immunostaining, HGT-1 cells on coverslips were pre-stained with 100 nM Mitotracker in DMEM without fetal calf serum (30 min, 37°C). After washes, HGT-1 cells were fixed (30 min, PBS, 4% paraformaldehyde) and then blocked and permeabilized with blocking solution (PBS, 2% Bovine Serum Albumin, 0.2% Triton X-100) during 1 h. Then, after washes, cells were incubated with sheep antibody raised against cytochrome c in blocking solution (dilution 1∶650, 2 h30). After washes, coverslips were incubated with anti-sheep IgG FITC-linked secondary antibody (dilution 1∶40) and with TO-PRO-3 (1 µM) diluted in blocking solution in the dark during 1 h.

After washing, cells were examined with a Nikon C1Si Laser Scanning Confocal Imaging System on inverted microscope TE2000-E (Nikon, Champigny-sur-Marne, France), equipped with an argon ion laser and two helium/neon lasers emitting respectively at 488, 543 and 633 nm.

### DNA fragmentation

HGT-1 cells were trypsinized, harvested and resuspended in lysis buffer (10 mM Tris-HCl pH 8, 10 mM EDTA, 1% Triton X100) containing 200 µg/ml of proteinase K. After incubation at 56°C for 2 h, RNA was digested by RNAse A (100 µg/mL) during 1 h at 37°C. Then, DNA was purified by phenol/chloroform extraction and precipitated by addition of 0.3 M sodium acetate and 2 volumes of cold ethanol as previously described [57]. The precipitates were centrifuged (12 000× *g*, 20 min, 4°C), and the pellets were air dried and resuspended in 10 mM Tris HCl pH 8 containing 1 mM EDTA. The DNA was quantified using a NanoDrop apparatus (Thermo scientific, Labtech, Palaiseau, France) according to the manufacturer's instructions. DNA fragmentation was analyzed by 1% agarose gel electrophoresis of DNA (3 µg) and staining with gelRed.

### Cell cycle analysis

HGT-1 cells were harvested by trypsinization, washed and incubated overnight with cold 70% ethanol in PBS prior to staining with a 40 µg/mL propidium iodide solution in PBS with 50 µg/mL RNase A (30 min, 37°C, in dark). 10 000 cells were analyzed for fluorescence emission (FL-3) by using a FACS flow cytometer FC500 (BD Biosciences) and the CXP software (BD Biosciences). This software was used to generate DNA content frequency histograms and quantify the amount of cells in the individual cell cycle phases including subG0/G1 population. Three independent experiments were carried out.

### Cell viability and apoptosis analysis by Annexin V/7-AAD staining

HGT-1 cells were trypsinized and harvested as described above. After washes in PBS, cells were stained with Annexin V-FITC and 7-AAD according to the manufacturer's instructions. Cells were then analyzed by flow cytometry. Cell analysis and data processing were carried out on 10 000 cells using the same device and software as above. Three independent experiments were performed.

### Reactive oxygen species detection

Production of ROS was assessed by flow cytometry using dihydroethidium (DHE) probe to detect superoxide anion (O_2_^−^) as previously described [60]. HGT-1 cells were harvested as described above, washed in PBS, and labeled with 5 µM DHE (15 min, 37°C, in the dark). Fluorescence emission (FL-1) of oxidized DHE was analyzed by flow cytometry for 10 000 cells. Menadione (100 µM, 20 min) was used as a positive control. Superposition of control and menadione histograms allowed defining a gate for calculating the percentage of cells accumulating O_2_^−^. Experiments were conducted in triplicate.

### Analysis of mitochondrial membrane potential (ΔΨm)

Disruption of ΔΨm was monitored by flow cytometry using 3,3′dihexyloxacarbocyanine iodide (DiOC6(3)) probe. HGT-1 cells were harvested as described above and washed with PBS, incubated with 50 nM DiOC6(3) (20 min, 37°C, in the dark). The decoupling agent carbonyl cyanide p-trifluoromethoxy-phenylhydrazone (FCCP; 50 µM, 20 min) served as a positive control for ΔΨm loss. Fluorescence emission (FL-1) was analyzed by flow cytometry or 10 000 cells. Superposition of control and FCCP histograms allowed to define a gate for calculating the percentage of cells with a decreased DiOC6(3) incorporation. Three independent experiments were carried out.

### Western blot analysis

HGT-1 cells were harvested as described above and cell pellets were stored at −80°C until extraction. Pellets were resuspended in lysis buffer (1% SDS, 10 mM DTT, protease inhibitor cocktail, 10 mM Tris-HCL pH 7.4) and sonicated. Cellular fragments were eliminated by centrifugation (11,000× g, 3 min, 4°C). To obtain a cytosol-enriched fraction, proteins were extracted as previously described [61]. Protein concentration of the total and cytosol-rich fractions was determined by the Bradford method. Proteins (50 µg) were separated on a polyacrylamide sodium dodecylsulfate (SDS)-containing gel and transferred to a PVDF membrane. After blocking nonspecific binding sites for 1 h at room temperature by 5% nonfat milk in TPBS (PBS with 0.1% Tween 20), membranes were incubated overnight at 4°C with specific antibodies. Then, membranes were washed twice with TPBS, incubated for 1 h at room temperature with horseradish peroxidase-conjugated goat anti-mouse or anti-rabbit Abs, washed twice with TPBS before revelation using an enhanced chemiluminescence detection kit (GE healthcare, Vélizy, France). Protein bands were visualized with a Syngene G:Box (Ozyme, Saint-Quentin-en-Yvelines, France).

### Measurement of caspase activities

Caspase-3 is the major effector (or executioner) caspase of apoptosis. Caspase-8 is an initiator caspase involved in the extrinsic (via death receptors) pathway of apoptosis. Caspase-9 is an initiator caspase involved in the intrinsic (via mitochondria) pathway of apoptosis [16]. Activity of the caspases -3, -8 and -9 was assessed as previously described [62]. Briefly, cell lysates were obtained and tested for their caspase activities using specific substrates. Caspase-3 activity was measured using the substrate Ac-DEVD-AMC, caspase-8 activity with Ac-IETD-AMC and caspase-9 activity with Ac-LEHD-AFC. Caspase activities were measured by monitoring fluorescence continuously in a dual luminescence fluorometer (Spectra max Gemini XS, Molecular Devices, Sunnyvale, CA, USA) using specific excitation and emission wavelengths for each peptide. Enzyme activity was determined as initial velocities and expressed as relative intensity (arbitrary unit)/min/µg protein. The experiments were performed in triplicates for each experimental condition.

### Statistical analysis

Results are expressed as the mean ± standard deviation (sd). Significance was determined by Student's t test for paired samples. The significance is shown as follows: *P<0.05; **P<0.01.

## Supporting Information

Figure S1 **Propionibacterial population and SCFAs production of 12 dairy propionibacteria strains in fermented milk.** Twelve dairy propionibacteria strains (supplemental Table S1) were cultivated during 3 days at 30°C. (**A**) Populations of dairy propionibacteria in fermented milks were determined by enumeration. (**B**) Production of short-chain fatty acids by dairy propionibacteria in fermented milks was determined by HPLC. (**A,B**) The results are means of at least two independent experiments.
(TIF)

Figure S2 **Kinetics of cell death induced by propionibacterial metabolite (A) or by camptothecin (B) is detected by 3 methods.** HGT-1 were treated as in Figure 1. Viability was monitored by methylene blue, MTT and trypan blue exclusion assays. Consistent results are obtained.
(TIF)

Figure S3 **Time course leading to 50% of HGT-1 cell death (T_1/2_) by fermented milk ultrafiltrate or fermented milk.** Time course leading to 50% of HGT-1 cell death (T_1/2_) was determined with the methylene blue assay described in Figure 1C. Results are mean values of three experiments ± sd.
(TIF)

Figure S4 **Camptothecin induces typical nuclear marks of apoptosis in human gastric cancer cells (positive control for** **Figure 2** **).** (**A**) Camptothecin-induced nuclear condensation. Cells were cultured (Co.) or treated for 24, 48 or 72 h with 4 µM camptothecin. Cells were then stained with Hoechst 33342 prior to fluorescence microscopy. Arrows indicate chromatin condensation (b), nuclear fragmentation (c) and formation of apoptotic bodies (d). (**B**) **C**amptothecin-induced DNA fragmentation in HGT-1 cells. Genomic DNA was extracted and analyzed in 1% agarose gel. HGT-1 cells were treated as in (A). (**C**) Camptothecin-induced changes in cell cycle phases. DNA content of HGT-1 cells was analyzed by flow cytometry after propidium iodide staining. Representative histograms corresponding to DNA content analysis of HGT-1 cells are shown. The percentage of the cell population with sub-G1 DNA content, indicative of apoptosis, is indicated. Proportion of each cell subsets (sub-G1, G0/G1, S, G2/M), within the total cell population, is shown for each time of treatment. Results are mean values of three independent experiments ± sd. *P<0.05, **P<0.01, treated cells versus control (0 h). The distribution of cell cycle phases of untreated cells (control) remained unchanged during the whole experiment.
(TIF)

Figure S5 **Camptothecin induces phosphatidylserine exposure at the plasma membrane outer leaflet in human gastric cancer cells (positive control for** **Figure 3** **).** Flow cytometry kinetic analysis of cell death in HGT-1 cells treated with 4 µM camptothecin. (**A**) A representative experiment of Annexin V/7-AAD staining of HGT-1 cells at each time of treatment is shown, with proportions of Annexin V positive cells (AV+; apoptotic cells). (**B**) Quantitative FACS analysis of Annexin V-FITC (AV) binding to HGT-1 cells was performed after counterstaining with 7-aminoactinomycin-D (7AAD). Presented values correspond to the proportion of each cell subsets, within the total cell population, for each treatment time. Results are mean values of three independent experiments ± sd. *P<0.05, **P<0.01, treated cells versus control (0 h).
(TIF)

Figure S6 **The ROS scavenger TEMPOL lowers propionibacterial metabolites-induced accumulation of anion superoxide (O_2_^−^).** HGT-1 cells were treated as in Figure 4C in the presence or absence of 5 mM TEMPOL. Flow cytometry analysis of anion superoxide (O_2_^−^) accumulation with DHE staining. (**A**) Overlays of a representative experiment of ROS detection. Cells were stained with dihydroethidium and analyzed by flow cytometry. The prooxidant menadione (Men., 100 µM, 15 min) was used as positive control. (**B**) Values are represented as a proportion of cells with increased ROS (increase of fluorescence intensity), within the total cell population, for each treatment. Results are mean values of three independent experiments ± sd. *P<0.05, **P<0.01, no TEMPOL versus TEMPOL.
(TIF)

Table S1 **Dairy propionibacteria strains and their origin.**
(DOC)

Table S2 **Dairy propionibacteria fermented milks induce apoptosis in human colorectal cells HT-29.**
(DOC)
