# Supplementary figures and images for: Milk Fermented by Propionibacterium freudenreichii Induces Apoptosis of HGT-1 Human Gastric Cancer Cells
Source: PLoS One. 2012 Mar 19;7(3):e31892. doi: 10.1371/journal.pone.0031892 (PMC3307715; doi:10.1371/journal.pone.0031892)

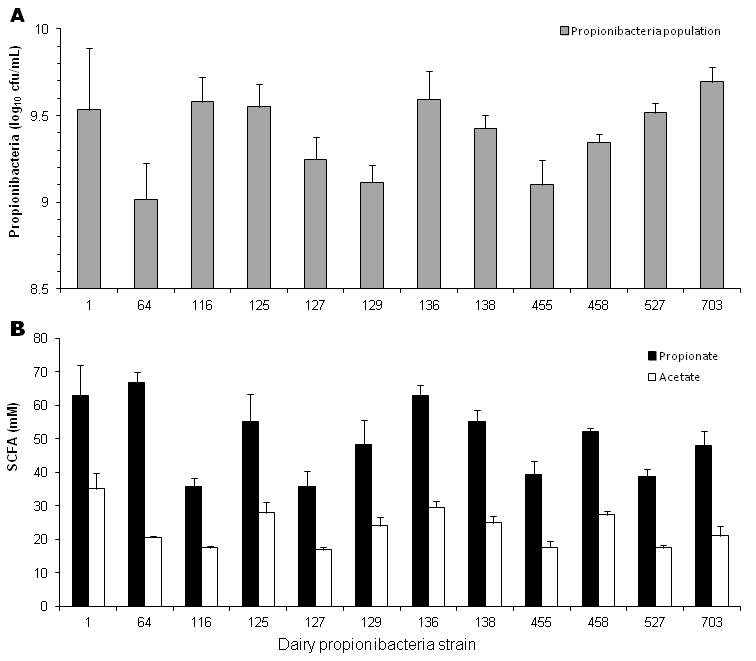

Supplement: Figure S1 — Propionibacterial population and SCFAs production of 12 dairy propionibacteria strains in fermented milk. Twelve dairy propionibacteria strains (supplemental Table S1) were cultivated during 3 days at 30°C. (A) Populations of dairy propionibacteria in fermented milks were determined by enumeration. (B) Production of short-chain fatty acids by dairy propionibacteria in fermented milks was determined by HPLC. (A,B) The results are means of at least two independent experiments. (TIF) [file pone.0031892.s001.tif]

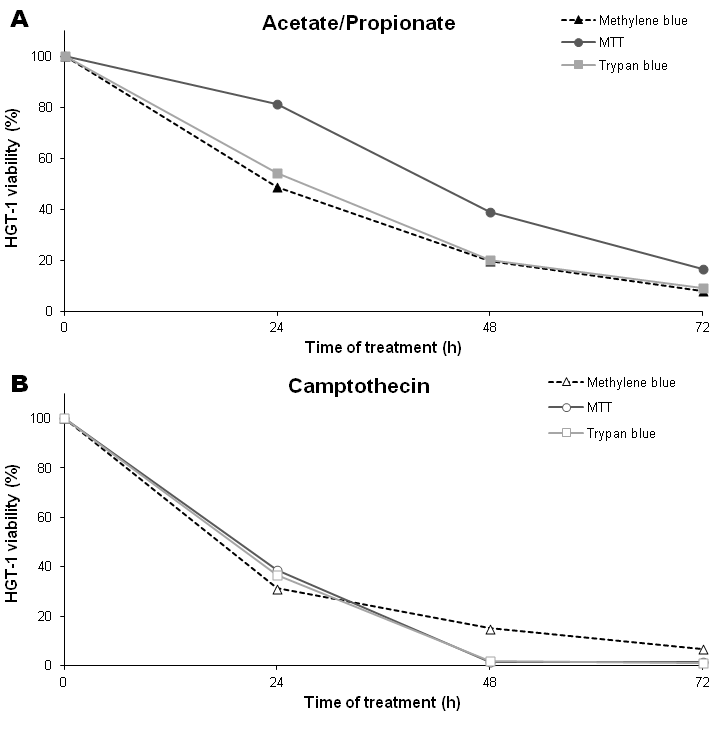

Supplement: Figure S2 — Kinetics of cell death induced by propionibacterial metabolite (A) or by camptothecin (B) is detected by 3 methods. HGT-1 were treated as in Figure 1. Viability was monitored by methylene blue, MTT and trypan blue exclusion assays. Consistent results are obtained. (TIF) [file pone.0031892.s002.tif]

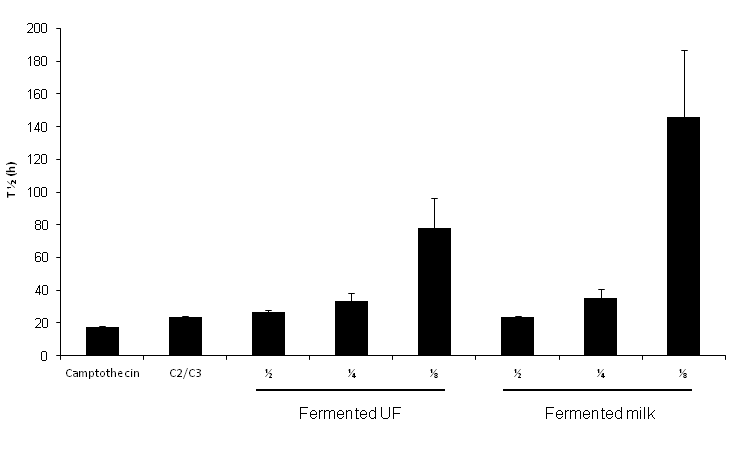

Supplement: Figure S3 — Time course leading to 50% of HGT-1 cell death (T1/2) by fermented milk ultrafiltrate or fermented milk. Time course leading to 50% of HGT-1 cell death (T1/2) was determined with the methylene blue assay described in Figure 1C. Results are mean values of three experiments ± sd. (TIF) [file pone.0031892.s003.tif]

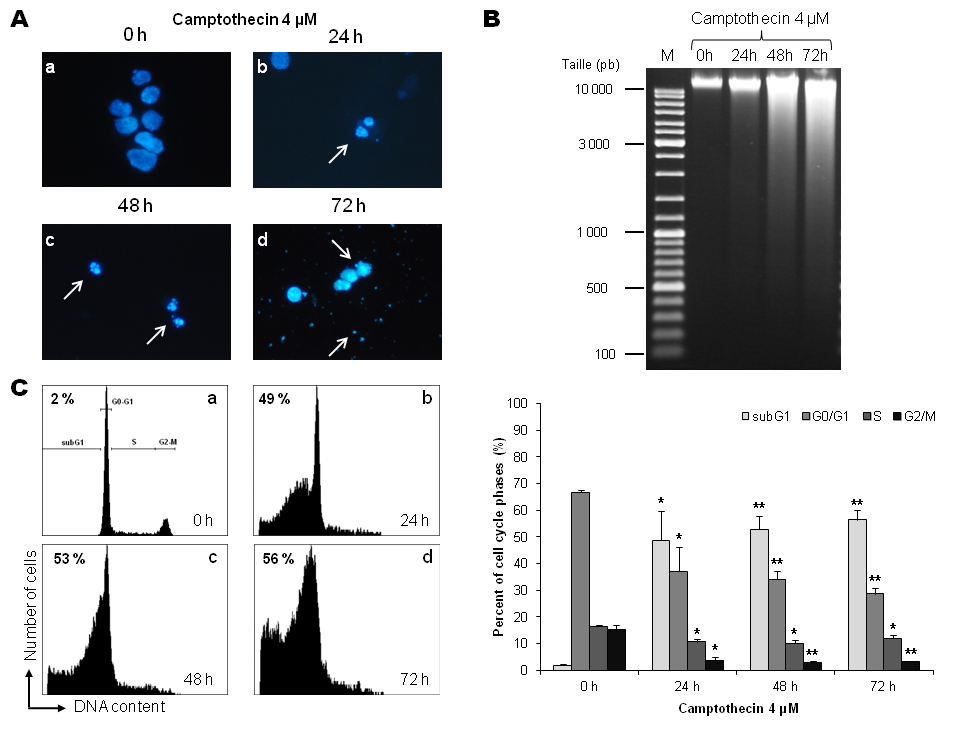

Supplement: Figure S4 — Camptothecin induces typical nuclear marks of apoptosis in human gastric cancer cells (positive control for Figure 2 ). (A) Camptothecin-induced nuclear condensation. Cells were cultured (Co.) or treated for 24, 48 or 72 h with 4 µM camptothecin. Cells were then stained with Hoechst 33342 prior to fluorescence microscopy. Arrows indicate chromatin condensation (b), nuclear fragmentation (c) and formation of apoptotic bodies (d). (B) Camptothecin-induced DNA fragmentation in HGT-1 cells. Genomic DNA was extracted and analyzed in 1% agarose gel. HGT-1 cells were treated as in (A). (C) Camptothecin-induced changes in cell cycle phases. DNA content of HGT-1 cells was analyzed by flow cytometry after propidium iodide staining. Representative histograms corresponding to DNA content analysis of HGT-1 cells are shown. The percentage of the cell population with sub-G1 DNA content, indicative of apoptosis, is indicated. Proportion of each cell subsets (sub-G1, G0/G1, S, G2/M), within the total cell population, is shown for each time of treatment. Results are mean values of three independent experiments ± sd. *P<0.05, **P<0.01, treated cells versus control (0 h). The distribution of cell cycle phases of untreated cells (control) remained unchanged during the whole experiment. (TIF) [file pone.0031892.s004.tif]

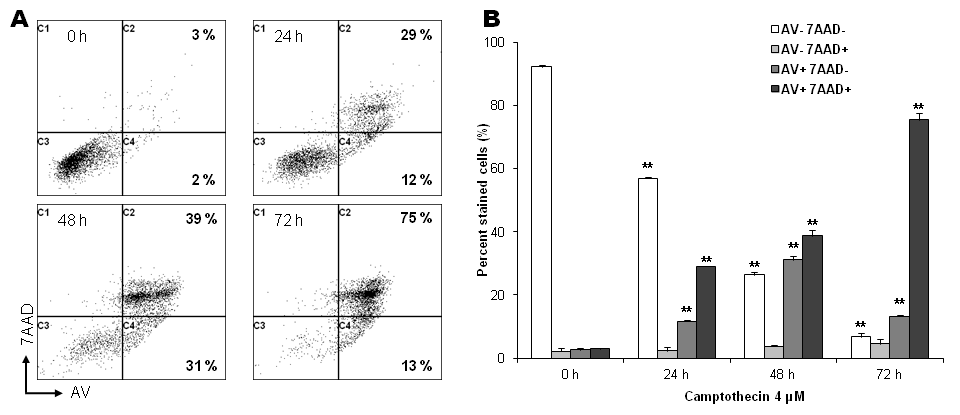

Supplement: Figure S5 — Camptothecin induces phosphatidylserine exposure at the plasma membrane outer leaflet in human gastric cancer cells (positive control for Figure 3 ). Flow cytometry kinetic analysis of cell death in HGT-1 cells treated with 4 µM camptothecin. (A) A representative experiment of Annexin V/7-AAD staining of HGT-1 cells at each time of treatment is shown, with proportions of Annexin V positive cells (AV+; apoptotic cells). (B) Quantitative FACS analysis of Annexin V-FITC (AV) binding to HGT-1 cells was performed after counterstaining with 7-aminoactinomycin-D (7AAD). Presented values correspond to the proportion of each cell subsets, within the total cell population, for each treatment time. Results are mean values of three independent experiments ± sd. *P<0.05, **P<0.01, treated cells versus control (0 h). (TIF) [file pone.0031892.s005.tif]

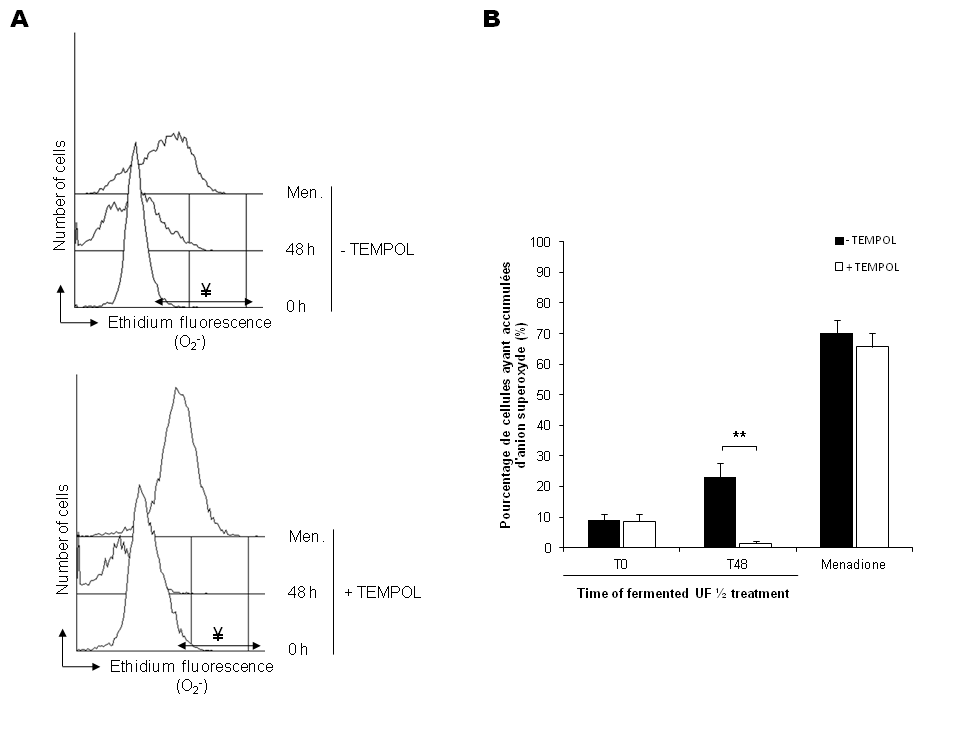

Supplement: Figure S6 — The ROS scavenger TEMPOL lowers propionibacterial metabolites-induced accumulation of anion superoxide (O2−). HGT-1 cells were treated as in Figure 4C in the presence or absence of 5 mM TEMPOL. Flow cytometry analysis of anion superoxide (O2 −) accumulation with DHE staining. (A) Overlays of a representative experiment of ROS detection. Cells were stained with dihydroethidium and analyzed by flow cytometry. The prooxidant menadione (Men., 100 µM, 15 min) was used as positive control. (B) Values are represented as a proportion of cells with increased ROS (increase of fluorescence intensity), within the total cell population, for each treatment. Results are mean values of three independent experiments ± sd. *P<0.05, **P<0.01, no TEMPOL versus TEMPOL. (TIF) [file pone.0031892.s006.tif]
