# Supplementary material for: Milk Fermented by Propionibacterium freudenreichii Induces Apoptosis of HGT-1 Human Gastric Cancer Cells
Source: PLoS One. 2012 Mar 19;7(3):e31892. doi: 10.1371/journal.pone.0031892 (PMC3307715; doi:10.1371/journal.pone.0031892)
Supplement: Table S1 — Dairy propionibacteria strains and their origin. (DOC) [file pone.0031892.s007.doc]

**Table S1: Dairy propionibacteria** strains and their origin

| Strain _a_ | | | |  | | Taxonomy | | | | |
| --- | --- | --- | --- | --- | --- | --- | --- | --- | --- | --- |
| CIRM | | Other name | |  | | Genus | | Species | | Subspecies |
| BIA1 | CIP103027 | |  | | *Propionibacterium* | | *freudenreichii* | | *shermanii* | |
| BIA64 | CNRZ80 | |  | | *Propionibacterium* | | *acidipropionici* | |  | |
| BIA116 | CNRZ81 | |  | | *Propionibacterium* | | *freudenreichii* | | *freudenreichii* | |
| BIA125 | ITG P14 | |  | | *Propionibacterium* | | *freudenreichii* | | *shermanii* | |
| BIA127 | ITG P18 | |  | | *Propionibacterium* | | *freudenreichii* | | *freudenreichii* | |
| BIA129 | ITG P20 | |  | | *Propionibacterium* | | *freudenreichii* | | *shermanii* | |
| BIA136 | IS | |  | | *Propionibacterium* | | *freudenreichii* | | *shermanii* | |
| BIA138 | ITG P9 | |  | | *Propionibacterium* | | *freudenreichii* | | *shermanii* | |
| BIA455 | CNRZ 87 | |  | | *Propionibacterium* | | *jensenii* | |  | |
| BIA458 | IS | |  | | *Propionibacterium* | | *freudenreichii* | | *shermanii* | |
| BIA527 | - | |  | | *Propionibacterium* | | *freudenreichii* | | *freudenreichii* | |
| BIA703 | IS | |  | | *Propionibacterium* | | *freudenreichii* | | *shermanii* | |

a Culture collections: CIRM-BIA, Centre International de Ressources Microbiennes - Bactéries d'Intérêt Alimentaire, INRA, Rennes ; CIP: Collection de l’Institut Pasteur, Paris, France ; CNRZ: Centre National de Recherche en Zootechnie, INRA, Jouy-en-Josas, France ; ITG: Institut Technique du Gruyère, Actilait, Rennes, France; IS: Industrial strains, Laboratoires Standa, Caen, France.
