# Supplementary material for: Milk Fermented by Propionibacterium freudenreichii Induces Apoptosis of HGT-1 Human Gastric Cancer Cells
Source: PLoS One. 2012 Mar 19;7(3):e31892. doi: 10.1371/journal.pone.0031892 (PMC3307715; doi:10.1371/journal.pone.0031892)
Supplement: Table S2 — Dairy propionibacteria fermented milks induce apoptosis in human colorectal cells HT-29. (DOC) [file pone.0031892.s008.doc]

**Table S2: Dairy propionibacteria fermented milks induce apoptosis in human colorectal cells HT-29**

| Treatment _a_ | |  | | HT-29 viability loss _b_ (%) | |  | | Caspase-3 activity _c_ (au/h/µg of proteins) |
| --- | --- | --- | --- | --- | --- | --- | --- | --- |
| *Controls* |  | |  | |  | |  | |
| Non fermented milk |  | | 9.72 ± 0.33 | |  | | 3.80 ± 0.55 | |
| Etoposide |  | | 67.82 ± 3.13 | |  | | 25.89 ± 1.54 | |
| C2/C3 |  | | 81.95 ± 1.47 | |  | | 15.38 ± 0.65 | |
| *Fermented milks* |  | |  | |  | |  | |
| BIA 1 |  | | 67,11 ± 2.87 | |  | | 5.97 ± 0.66 | |
| BIA 64 |  | | 65,22 ± 0.35 | |  | | 104.15 ± 6.64 | |
| BIA 116 |  | | 62,49 ± 1.36 | |  | | N.D. | |
| BIA 138 |  | | 78,01 ± 2.72 | |  | | 44.28 ± 2.62 | |
| BIA 455 |  | | 60,83 ± 0.86 | |  | | N.D. | |

a HT-29 cells were treated during 48 h with a ½ dilution, in DMEMc, of supernatants obtained from five fermented milks. As negative control, cells were treated with ½ dilution of non fermented milk. As positive controls, cells were treated with DMEMc containing a mixture of acetate and propionate (C2/C3, 15/30 mM) or etoposide (100 µM).

b HT-29 viability loss was determined by methylene blue assay and calculated as described in materials and methods. Results are mean values of three experiments ± sd.

c Caspase-3 specific activity of lysates from cells treated as above was studied using indicated peptides and calculated as described in materials and methods. Results are mean values of three experiments ± sd.
